# The Perception of the Severity of Facial Asymmetry among Laypersons, General Practitioners, Orthodontists, and Maxillofacial Surgeons

**DOI:** 10.30476/DENTJODS.2020.84790.1103

**Published:** 2021-06

**Authors:** Nazgol Zamanian, Alireza Jafari-Naeimi

**Affiliations:** 1 Dept. of Orthodontics, Faculty of Dentistry, Tehran Medical Sciences, Islamic Azad University, Tehran, Iran

**Keywords:** Facial asymmetry, Three-dimensional images, Perception, Orthodontics

## Abstract

**Statement of the Problem:**

The degree of asymmetry perception of dental and medical practitioners is influenced by several factors. The perceived asymmetry affect the treatment plan design.

**Purpose:**

The aim of the present study was to investigate the consistency of facial asymmetry and identify the amounts of transverse asymmetry that can be regarded as normal and might need correction.

**Materials and Method:**

In this cross-sectional descriptive study, three-dimensional (3D) images of a man and a women volunteer were obtained. Then transverse changes were applied by ZBrush software so that for each volunteer, seven 3D images of their face with varying degrees of facial transverse asymmetry were created. Then, the images were displayed to four groups of observers including layperson, general dentists, orthodontists, and maxillofacial surgeons. Finally, the consistency of the perception of these four groups of observers with the different degrees of facial asymmetry was compared

**Results:**

Fourteen photographic samples were evaluated and ranked by 80 observers in four groups. The consistency of the perception of the facial transverse asymmetry was equal to 33%, which indicated a lack of consistency.

**Conclusion:**

According to the findings of this study, there was no consistency between the groups. The perception of dental professionals and ordinary people regarding the severity of transverse facial asymmetry seems to be inconsistent.

## Introduction

Symmetry is a fundamental geometry property that influences people’s aesthetic experience in familiar ways in different cultures and historical periods, but the origins of this global predilection for symmetrical patterns is ambiguous [ 1]. Facial asymmetry is about equality in size, shape, and fit of features on both sides of the midsagittal plane [ 2]. One of the most widely studied facial features among orthodontists is bilateral facial symmetry. Facial symmetry plays an essential role in the attractiveness of the face and its acceptance in society [ 3]. Severe facial malformations can be a manifestation of the craniofacial syndrome, trauma, pathology, and abnormal growth that can have significant psychological, functional, and aesthetic consequences for patients as well as affecting self-esteem and quality of life [ 4- 5]. On the other hand, Patcas *et al*. [ 6] showed that orthognathic treatments that also include correction of facial asymmetries have a beneficial effect on attractiveness in 74% of patients.

The degree of perceived asymmetry of the patient's face by the dental and medical practitioners is useful in determining the severity of the facial asymmetry and designing an effective treatment plan [ 7- 9]. It has been suggested that gender, culture, and ethnicity may influence practitioner perceptions towards facial asymmetry [ 10- 11]. Most cases of facial asymmetry do not essentially point out considerable structural or functional problems. Patients seek treatments because of the impairment in their facial attractiveness [ 12]. Chu *et al*. showed that there should be at least 3 mm of facial asymmetry in the digitally manipulated image for an average person to recognize asymmetry [ 13]. Most studies in the case of the facial asymmetry perception have used two-dimensional photos [ 14] and some of them used typical individual images rather than fully mirrored images [ 15].

The aim of the present study was to evaluate the perceptions of the facial symmetry among laypersons, general dental practitioners, orthodontists and oral and maxillofacial surgeons using manipulated 3D photographs.

## Materials and Method

The ethical approval of the present study was received from the Islamic Azad University Research and Ethics Committee.

### Image Synthesis Deformation Simulation

First, a male and a female volunteer were selected, and they were photographed in a 3D scan room using a DSLR Canon (Canon Inc., Tokyo, Japan) camera. Then by an anatomy modeler artist, using ZBrush software (Pixologic, Inc., Los Angeles, USA), these images were modified in the transverse dimension so that there were seven models for each person's image. In the first mode, the person's image was completely symmetric. In other words, by using a mirror image option and moving one side of the face to the other and created an utterly symmetric image of the frontal view. Then, using the software, we created the transverse facial asymmetry as follows. We created 1mm transverse asymmetry on the right side, 2mm transverse asymmetry on the left side, 3 mm transverse asymmetry on the right side, 5 mm transverse asymmetry on the left side of the face, 6 mm transverse asymmetry on the right side, and 8 mm transverse asymmetry on the left side. The midsagittal line was considered as the reference line to manipulate images and to create the transverse asymmetries. Then the nose, lip, chin, and mandibular angle were moved using ZBrush software to create varying degrees of asymmetry from 1 mm to 8 mm.

### Asymmetry Perception Assessment

**Figure 1:**
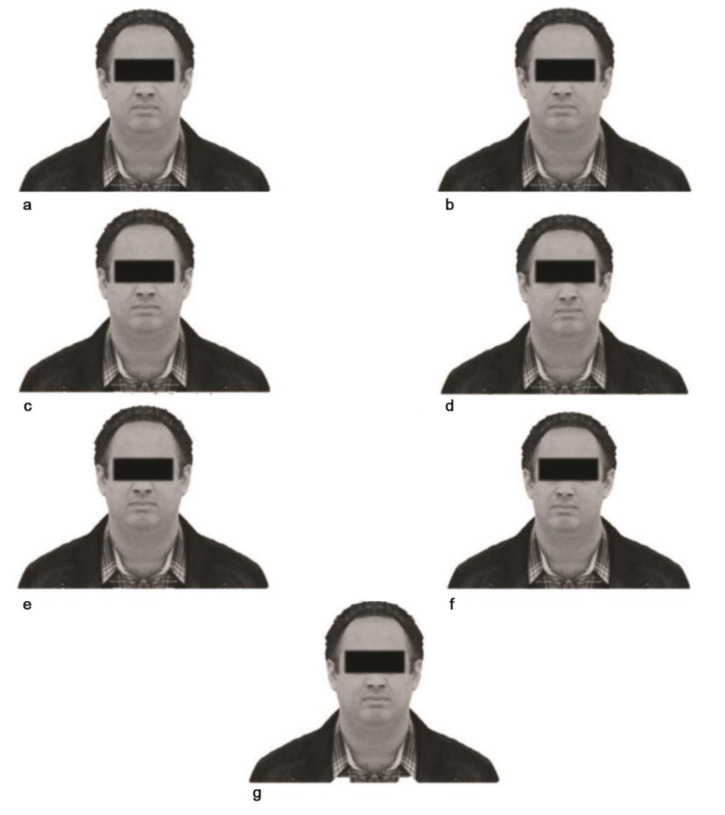
Examples of male volunteer faces with various levels of asymmetry. **a:** symmetric face; **b:** 4mm asymmetry to the right; **c:** 1mm asymmetry to the left; **d:** 6mm asymmetry to the left; **e:** 2mm asymmetry to the right; **f:** 8mm asymmetry to the right; **g:** 3mm asymmetry to the left

The frontal view of the volunteers was used to assess the severity of facial asymmetry. We had seven photographs of each volunteer (Figure 1) with various transverse facial asymmetries. 

The 3D images were randomly presented to each of the four groups of observers (n = 20), including laypersons, general dental practitioners, orthodontists, and oral and maxillofacial surgeons, within a limited time (10 seconds). We asked the participants to determine the magnitude of asymmetry in each image based on the three options in the questionnaire (“completely symmetrical”“, non-symmetrical but aesthetically acceptable”“, non-symmetrical and requiring treatment”). Then, the responses of these four groups of observers on the extent of the transverse facial deformity were compared. 

For the assessment of the accuracy of each group responses, we considered the responses of the observers to be “completely symmetrical” in the 0 to 1mm interval, “non-symmetrical, but aesthetically acceptable”, in the 2 to 4mm interval, and “non-symmetrical and requiring treatment” in the 6 to 8 mm interval to be correct answers.

### Statistical Analysis

The rate of compliance was defined in three categories as acceptable (above 75%), moderate (between 40-75%), and non-compliance (below 40%). In the present study, we used Pearson's chi-squared test for comparing study groups using Statistical Package for the Social Sciences software version 19 (Chicago, `IL, USA). A P-value of less than 0.05 was considered significant.

## Results

Fourteen photographic images were taken, and then 80 individuals in four groups (including orthodontists, maxillofacial surgeons, general dentists, and laypersons) assessed the severity of transverse asymmetry. The subjects included 50 men and 30 women with a mean age of 34 ± 8.59 years.

### Reliability Analysis

For reliability analysis, the subjects’ answers to the questionnaire were compared (Table 1). Our findings showed that the consistency of transverse facial asymmetry diagnosis among all groups was equal to 33 percent. As it was less than 40 percent, it can be concluded there was an inconsistency among the observers answer.

**Table1:** Comparison of facial asymmetry perception between the observer groups

	Symmetric	Partially Symmetric	Asymmetric
Laypersons	562	568	550
Dentists	526	541	613
Surgeons	514	526	640
Orthodontists	550	568	562

### Perceived Asymmetry Among Each Group

**Figure 2:**
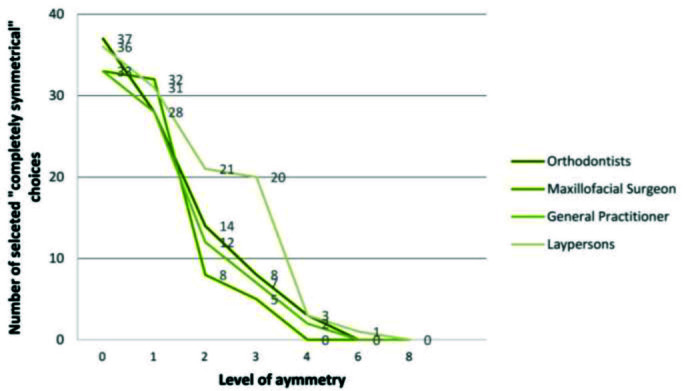
The total number of “completely symmetrical” choice selection of various groups of observers at different intensities of symmetry.

Laypersons chose “completely symmetrical” for images with varying intensities of transverse asymmetry more than any other groups. On the other, maxillofacial surgeons selected “completely symmetrical” less than other groups. There was a statistically significant difference between the laypeople and the maxillofacial surgeons in choosing this option (*p*= 0.003). The highest number of “non-symmetrical and requiring treatment” option selection belonged to the maxillofacial surgeons, and the lowest number belonged to laypeople. However, there was no significant difference in among various groups (Table 1). The selection of “completely symmetrical” choice of various groups of observers at different intensities of asymmetry is given in Figure 2. In case of a perfectly mirrored image, the most accurate diagnosis of facial symmetry belonged to orthodontists. At the interval of 0 mm to 3 mm asymmetry, more than half of laypersons did not report any asymmetry. However, when asymmetry reached 4 mm, laypeople could detect asymmetry like other study groups.

### The Effect of patient Gender in the Perception of Asymmetry

**Figure 3:**
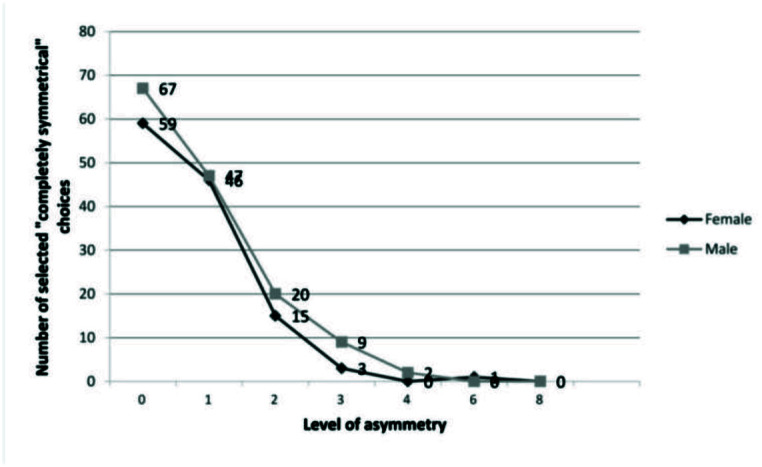
The total number of “completely symmetrical” choice selection from various genders of manipulated images at different intensities of asymmetry.

The selection of “completely symmetrical” choice at different intensities of asymmetry was used to test for differences in perception according to the gender of the volunteer’s manipulated photos (Figure 3). Although there was a slight difference between asymmetry perception in case of gender, there were no statically significant differences.

### Evaluation of Observers’ Responses

According to Table 2, general dentists gave the least number of correct answers in the interval of 0 to 1 mm. In the case of 2 to 4 mm asymmetry, orthodontist had the highest number of correct answers, and laypeople had the highest number of wrong answers. Furthermore, it has been observed that maxillofacial surgeons were most likely to treat these patients. Finally, in 6 to 8 mm interval, all groups answered correctly in 91 to 97.5 percent of cases.

**Table2:** Number of correct answers in various groups in different intervals of asymmetry

Study Group		Laypersons	Dentists	Surgeons	Orthodontists
0 mm to 1 mm Transverse Asymmetry	Correct Answers	67 (83.5%)	61 (76%)	65 (81%)	65 (81%)
	Wrong Answers; “non-symmetrical but aesthetically acceptable”	12 (15%)	19 (24%)	14 (17.5%)	15 (19%)
	Wrong Answers; “non-symmetrical and requiring treatment”	1 (1.5%)	0	1 (1.5%)	0
2 mm to 4 mm Transverse Asymmetry	Correct Answers	61 (76%)	63 (52.5%)	66 (55%)	77 (64%)
	Wrong Answers; “completely symmetrical”	44 (36.5%)	21 (17.5%)	13 (11%)	25 (20%)
	Wrong Answers; “non-symmetrical and requiring treatment”	15 (12.5%)	36 (30%)	41 (34%)	18 (16%)
6 mm to 8 mm Transverse Asymmetry	Correct Answers	73 (91%)	75 (94%)	78 (97.5%)	76 (95%)
	Wrong Answers; “completely symmetrical”	1 (1.5%)	0	0	0
	Wrong Answers; “non-symmetrical but aesthetically acceptable”	6 (7.5%)	5 (6%)	2 (2.5%)	4 (5%)

## Discussion

Facial asymmetry means inequalities on both sides of the midsagittal axis that affects the attractiveness of individuals and leads to functional problems. Congenital disorders, acquired diseases, trauma, and developmental deformities can cause facial asymmetry [ 3]. Since there is no specific standard for the diagnosis of facial asymmetry [ 16], the present study aimed to present such information to the treatment team, including maxillofacial surgeons and orthodontists. 

In this study, three-dimensional photographic images were used to assess the perception of transverse facial asymmetries. The use of 3D images of male and female volunteers while preserving the natural color and structure of the face made the modeling to be closer to the actual face of the person. Given that each person's face naturally has various degrees of asymmetry [ 17], the mirror imaging option was used in this study to move one side of the volunteer face to the other and create a complete mirror image for the samples.

This study showed that there were no significant differences in the perception of transverse asymmetry between laypersons, general dentists, and orthodontists. However, we concluded that the perception of dentists and ordinary people about the severity of asymmetry is inconsistent. Our results are consistent with Meyer-Marcotty *et al*. study [ 15], which suggested there was no significant difference in the perception of facial asymmetry by dental professionals and the laypersons. However, Bispo de Carvalho Barbosa *et al*. [ 11] suggested that laypersons are less sensitive in the perception of facial asymmetry. In case of accuracy of perception, similar to previous studies conducted by Jarosz *et al*. [ 18], Pinho *et al*. [ 19], and Kokich *et al*. [ 20], orthodontists were the most accurate group in detecting various degrees of transverse asymmetry. Dong *et al*. [ 21] used 3D images to assess the influence of chin asymmetry on perceived facial esthetic among orthodontists, general dentists, and laypersons and suggested that the reason for the orthodontists to be the most accurate group is that they receive rigorous training and have more diagnostic experience. Therefore, the professionals know where to focus and more likely to focus on details [ 21].

In this study, 3D images of a male face and a female face were made to detect facial asymmetry in the nose and chin. McAvinchey *et al*. [ 7] conducted a similar study in the diagnosis of chin asymmetry. In their study, orthodontists showed the most sensitivity to diagnosis of asymmetry; there was a significant difference in diagnosis of asymmetry between dentists and the laypeople. However, the manipulation of the images was just on the chin region, which is a reason for their findings to be inconsistent with the results obtained in our study.

In addition, Jarosz *et al*. [ 18] used an online website for their survey on detecting chin asymmetry, which allowed survey takers to respond on mobile devices, tablets, and home computers. These equipments have varying monitor sizes, resolution and brightness, all of which could have affected the visual interpretations and perceptions of the chin asymmetries. In our study, we have controlled this issue by using a standardized digital display and all of our survey takers observe the photographs on the same device.

Another outcome of the current study was the difference in the perception of transverse facial asymmetry between laypersons and maxillofacial surgeons. The main reason for this difference in understanding can be the experience and knowledge of maxillofacial surgeons compared to laypeople. Another reason may be that each person's face is generally one of the revealing parts of his body. Most people focus their attention on the central part of the face (eyes) at first glance to the new individual face [ 17]. Another finding in the present study was that orthodontists were more likely to choose asymmetric but aesthetically acceptable option compared to surgeons, indicating that this group is acting more conservative in the case of facial asymmetry. However, maxillofacial surgeons chose the need of surgery option more than other groups. This finding indicates that this group is willing to undergo surgical intervention regardless of the apparent malformation. A probable reason might be their profession and higher standards for facial esthetics, which make them, prefer surgical treatments even when the asymmetry is esthetically acceptable.

Other factors that were considered in the diagnosis of facial asymmetry were the gender of the patients. It has been reported that various factors can influence the perception of transverse facial asymmetry, including the level of observers’ education and their gender [ 7]. Our study showed that there was no significant difference between male and female patients. McAvinchey *et al*. [ 7] also showed similar results concerning the gender of the patients.

Though an assessment of asymmetry in the transverse plane was performed here, the asymmetry in vertical plane, the role of distribution of other structures of the face like nose versus chin or eye asymmetries may affect the perception of overall asymmetry, deserving further evaluation. In a recent work by Chou *et al*. [ 22], a panel of young children as observers was included in the study. Interestingly, the results showed that pre-adolescent raters presented a similar or higher perception of facial asymmetry than adult raters. In such studies in the future, it is important to respect and to consider other categories of professionals and lay persons especially those involved with treating or affected by facial asymmetries as the group of observers. 

## Conclusion

The results of the current study imply that the perception of dentists and ordinary people about the severity of transverse facial asymmetry appears to be inconsistent. This study was performed based on images and manipulated models, and they cannot be a complete replacement for the actual face condition, and muscles play an essential role in this case, that requires further researches.

## References

[1] Huang Y, Xue X, Spelke E, Huang L, Zheng W, Peng K ( 2018). The aesthetic preference for symmetry dissociates from early-emerging attention to symmetry. Sci Rep.

[2] Peck S, Peck L, Kataja M ( 1991). Skeletal asymmetry in esthetically pleasing faces. Angle Orthod.

[3] Grammer K, Thornhill R ( 1994). Human (Homo sapiens) facial attractiveness and sexual selection: the role of symmetry and averageness. J Comp Psychol.

[4] Macgregor FC ( 1970). Social and psychological implications of dentofacial disfigurement. Angle Orthod.

[5] Rumsey N, Clarke A, White P, Wyn-Williams M, Garlick W ( 2004). Altered body image: appearance-related concerns of people with visible disfigurement. J Adv Nurs.

[6] Patcas R, Bernini DAJ, Volokitin A, Agustsson E, Rothe R, Timofte R ( 2019). Applying artificial intelligence to assess the impact of orthognathic treatment on facial attractiveness and estimated age. Int J Oral Maxillofac Surg.

[7] McAvinchey G, Maxim F, Nix B, Djordjevic J, Linklater R, Landini G ( 2014). The perception of facial asymmetry using 3-dimensional simulated images. Angle Orthod.

[8] Monnet-Corti V, Antezack A, Pignoly M ( 2018). Perfecting smile esthetics: keep it pink. Orthod Fr.

[9] Chisini LA, Noronha TG, Ramos EC, Dos Santos-Junior RB, Sampaio KH, Faria ESAL, et al ( 2019). Does the skin color of patients influence the treatment decision-making of dentists? A randomized questionnaire-based study. Clin Oral Investig.

[10] Little AC, Burt DM, Penton-Voak IS, Perrett DI ( 2001). Self-perceived attractiveness influences human female preferences for sexual dimorphism and symmetry in male faces. Proc Biol Sci.

[11] Bispo de Carvalho Barbosa P, de Andrade Vieira W, de Macedo Bernardino I, Costa MM, Pithon MM, Paranhos LR ( 2019). Aesthetic facial perception and need for treatment in simulated laterognathism in male faces of different ethnicities. Oral Maxillofac Surg.

[12] Lee SF, Dumrongwongsiri S, Lo LJ ( 2019). Perception of Lip Cant as a Sign of Facial Deformity: Assessment by Laypersons and Professionals on Composite Face Photographs. Ann Plast Surg.

[13] Chu EA, Farrag TY, Ishii LE, Byrne PJ ( 2011). Threshold of visual perception of facial asymmetry in a facial paralysis model. Arch Facial Plast Surg.

[14] Naini FB, Donaldson AN, McDonald F, Cobourne MT ( 2012). Assessing the influence of asymmetry affecting the mandible and chin point on perceived attractiveness in the orthognathic patient, clinician, and layperson. J Oral Maxillofac Surg.

[15] Meyer-Marcotty P, Stellzig-Eisenhauer A, Bareis U, Hartmann J, Kochel J ( 2011). Three-dimensional perception of facial asymmetry. Eur J Orthod.

[16] Hohman MH, Kim SW, Heller ES, Frigerio A, Heaton JT, Hadlock TA ( 2014). Determining the threshold for asymmetry detection in facial expressions. Laryngoscope.

[17] Mertens I, Siegmund H, Grusser OJ ( 1993). Gaze motor asymmetries in the perception of faces during a memory task. Neuropsychologia.

[18] Jarosz KF, Bosio JA, Bloomstein R, Jiang SS, Vakharia NS, Cangialosi TJ ( 2018). Perceptions of chin asymmetries among dental professionals and laypersons. Am J Orthod Dentofacial Orthop.

[19] Pinho S, Ciriaco C, Faber J, Lenza MA ( 2007). Impact of dental asymmetries on the perception of smile esthetics. Am J Orthod Dentofacial Orthop.

[20] Kokich VO, Kokich VG, Kiyak HA ( 2006). Perceptions of dental professionals and laypersons to altered dental esthetics: asymmetric and symmetric situations. Am J Orthod Dentofacial Orthop.

[21] Dong T, Ye N, Yuan L, Wu S, Xia L, Fang B ( 2020). Assessing the Influence of Chin Asymmetry on Perceived Facial Esthetics With 3-Dimensional Images. J Oral Maxillofac Surg.

[22] Chou PY, Denadai R, Chen SH, Tseng HJ, Hsu CK, Wang SW, et al ( 2019). Identifying Three-Dimensional Facial Fluctuating Asymmetry in Normal Pediatric Individuals: A Panel Assessment Outcome Study of Clinicians and Observers. Journal of Clinical Medicine.

